# LncRNA-MCM3AP-AS1 Promotes the Progression of Infantile Hemangiomas by Increasing miR-138-5p/HIF-1α Axis-Regulated Glycolysis

**DOI:** 10.3389/fmolb.2021.753218

**Published:** 2021-09-29

**Authors:** Haijun Mei, Hua Xian, Jing Ke

**Affiliations:** Department of General Surgery, Affiliated Hospital of Nantong University, Nantong, China

**Keywords:** LncRNA-MCM3AP-AS1, miR-138-5p, HIF-1α, infantile hemangiomas, glycolysis

## Abstract

Infantile hemangioma (IH) is a common benign tumor of endothelial cells in infants. Most hemangiomas are self-limited, but a few may develop and lead to serious complications that affect the normal life of children. Therefore, finding an effective treatment strategy for IH is a pressing need. Recent studies have demonstrated that non-coding RNAs affect the progression of multiple tumors. This study aims to investigate the mechanism by which LncRNA-MCM3AP-AS1 promotes glycolysis in the pathogenesis of IH. We first documented that the expression of LncRNA MCM3AP-AS1 was significantly upregulated in IH. Furthermore, we demonstrated that MCM3AP-AS1 bound to miR-106b-3p which promotes glycolysis in IH. In addition, we found that inhibition of HIF-1α contributed to the transformation of glycolysis to normal aerobic oxidation, partially reversed the promoting effect on glycolysis by the up-regulation of LncRNA MCM3AP-AS1 in IH disease. More importantly, we demonstrated this phenomenon existed in IH patients. Taken together, we demonstrate that LncRNA-MCM3AP-AS1 promotes the progression of infantile hemangiomas by increasing the glycolysis via regulating miR-138-5p/HIF-1α axis.

## Introduction

Infantile hemangiomas (IH) is a common benign skin tumor in children with vascular endothelial cell proliferation as the main pathological characteristic (Chamli et al., 2021). The incidence of IH is about 10% in Caucasians under 1 year old, and it is most common in females, with a male to female ratio of 1:3–4 (Chang et al., 2008). Potential complications of IH include permanent disfigurement, ulcers, scarring, bleeding, visual impairment, airway obstruction, congestive heart failure and death (Baselga et al., 2016). Compared with localized IH lesions, deep lesions are at greater risk of ulceration and dysfunction, and therefore require early and aggressive clinical intervention and treatment (Cheng and Friedlander, 2016). According to the course of disease, IH can be divided into proliferating phase stage, involuting stage and involuted stage (Yoon et al., 2021). Hemangiomas can recede spontaneously, but 90% of IH patients need 9–10 years or more to recede completely (Darrow et al., 2015). Most of the subsided tumors will leave scar or fibrous fat deposition, therefore early active intervention and treatment would be preferred. The tendency of spontaneous regression is an important feature of the course of IH, and its pathological basis is the disappearance of juvenile capillary degeneration, replaced by deposition of fiber and adipose tissues (Caussé et al., 2013). Promoting the transition from early stage to regression stage is the main goal of current treatment. However, due to the unknown mechanism of progression of infantile hemangioma, especially the mechanism of spontaneous regression, no effective treatment is available.

Long non-coding RNAs (lncRNAs) are a group of RNAs over 200 bp in length that do not have complete protein coding function and lack a specific open reading frame (Ørom and Shiekhattar, 2011). Previous studies have shown that lncRNAs affect the progression of multiple tumors (Ørom and Shiekhattar, 2011). LncRNA antisense 1 to micro-chromosome maintenance protein 3-associated protein (MCM3AP-AS1) gene was located on chromosome 21. In recent years, it has been reported that MCM3AP-AS1 plays an important role in the progression of glioblastoma and liver cancer (Yang et al., 2017; Wang et al., 2019a). Reports have shown that LncRNA MCM3AP-AS1 is up-regulated in glioma endothelial cells and hepatocellular carcinoma. Interference or silencing of its expression inhibits the proliferation and invasion of glioma cells. In papillary thyroid carcinoma, MCM3AP-AS1 expression is upregulated and promotes proliferation, migration, and invasion of cancer cells. However, the role of lncRNA MCM3AP-AS1 in hemangioma remains unknown.

MicroRNAs (miRNAs) are non-coding RNA molecules with highly conserved sequences (Dong et al., 2013). MiRNAs are involved in the regulation of a series of physiological processes, including cell proliferation, differentiation, apoptosis, signal transduction and organ development (Sarkar and Kumar, 2021). In prostate cancer, miRNA-34c down-regulates Bcl-2 protein expression, thereby inhibiting cell proliferation and promoting cell apoptosis (Hagman et al., 2010). In glioblastoma, miRNA-153 promotes apoptosis by down-regulating Bcl-2 protein expression (Xu et al., 2011). However, it is not clear whether miR-138-5p plays a role in hemangioma.

Due to the infinite proliferation of malignant tumor cells, their demand for energy and biomacromolecules increases dramatically (Zhang et al., 2017). The Warburg effect is known as the preferential conversion of glucose to lactic acid (aerobic glycolysis) for energy in cancer cells, even when oxygen is available (Wan et al., 2017). Interestingly, the vascular endothelial cells produce most of their ATP through aerobic glycolysis, despite that hemangioma contains abundant oxygen (Wang et al., 2019b). A variety of genes are often altered by changing their metabolic patterns during the process of tumor cells adapting to hypoxia. Among them, the most important gene is hypoxia-inducible factor 1a (HIF-1α) (Nyga et al., 2019). Under hypoxic conditions, HIF-1α promotes the occurrence of glycolysis in tumor cells by activating the expression of key protein involved in extracellular glucose input such as glucose transporter 1 (GLUT1) and intracellular glycolytic enzymes such as phosphofructokinase 1 (PFK1) (Quiroga et al., 2021; Zhang et al., 2021). Therefore, the therapeutic value of the glycolysis pathway in the pathogenesis of IH should be investigated.

In this study, we used bioinformatics method, combined with qPCR, WB, flow cytometry and other research methods to study the regulation of LncRNA MCM3AP-AS1 and miRNA/HIF-1α signaling pathway in the occurrence and development of hemangioma to explore the molecular mechanism of hemangioma.

## Results

### LncRNA MCM3AP-AS1 Expression was Up-Regulated in IH Clinical Samples and HemECs, and Affected the Prognosis of IH

**FIGURE 1:**
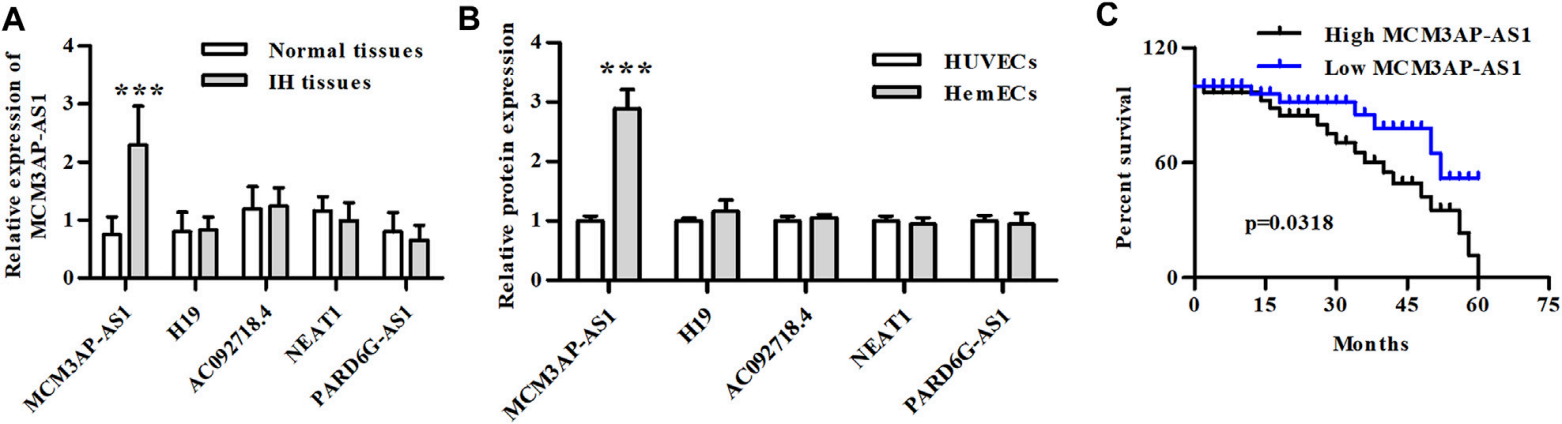
The relative mRNA expression of LncRNAs in IH clinical samples and IH cells. **(A)** The relative mRNA expression of LncRNAs in IH patients (***p < 0.001, compared with the Normal tissue group). **(B)** The relative mRNA expression of LncRNAs in HemECs and HUVECs cell lines (***p < 0.001, compared with the HUVECs group). **(C)** Kaplan-Meier analysis of OS compared high- and low-expression of LncRNA MCM3AP-AS1 in IH patients.

The qPCR was used to detect the expression of common lncRNAs to investigate their differential expression in IH and normal tissues. The results showed that among a variety of lncRNAs, only LncRNA MCM3AP-AS1 was up-regulated in IH compared to normal tissue (Figure 1A). We then compared the expression of these lncRNAs in hemangioma cell lines and normal cell lines, and our experimental results demonstrated that the expression of LncRNA MCM3AP-AS1 was significantly up-regulated in HemECs compared to the HUVECs that was taken as controls (Figure 1B). Since LncRNA MCM3AP-AS1 was up-regulated in both IH tissues and HemECs, the data from the SEER database were used to analyze whether LncRNA MCM3AP-AS1 affects the prognosis of hemangioma patients. The result indicated that patients with low expression of LncRNA MCM3AP-AS1 had significantly better overall survival (Figure 1C). These results suggest that the overexpression of lncRNA MCM3AP-AS1 aggravates the progression of IH.

### Knockdown of LncRNA MCM3AP-AS1 Inhibited the Proliferation of Hemangioma Cells

**FIGURE 2:**
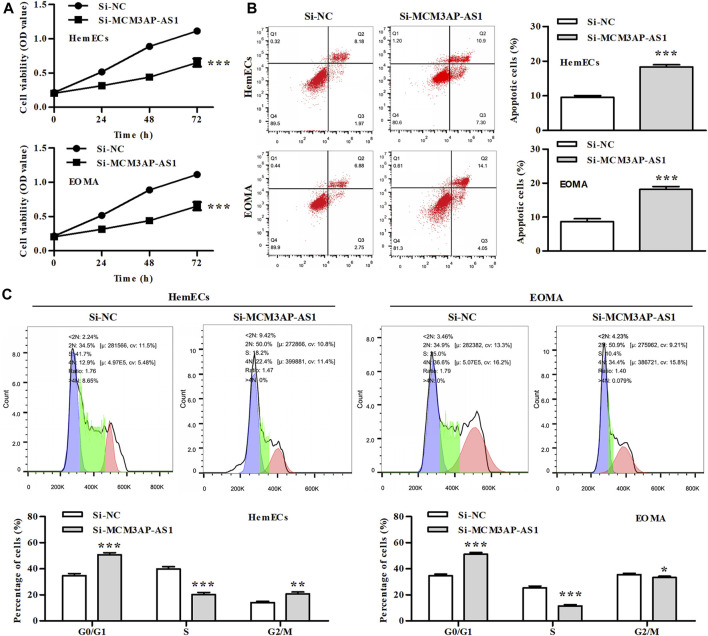
The effect of silencing LncRNA MCM3AP-AS1 on proliferation of IH cells. **(A)** In CCK-8 assay, cell viability was presented by OD value after HemECs cells were transfected with siNC mimics or si-MCM3AP-AS1 24, 48 and 72 h. **(B)** FACS analysis showing the apoptosis induced by siNC mimics or siMCM3AP-AS1 in HemECs. **(C)** Cell cycle analysis showing siMCM3AP-AS1 induced HemECs cell cycle arrest at the G1 phase.*p < 0.05, **p < 0.01, ***p < 0.001, compared with the si-NC group.

Previous research has reported the tumor-promoting effect of LncRNAs on several tumors. We explored whether LncRNA MCM3AP-AS1 has the similar effect on IH cells. CCK-8 analysis demonstrated that the cell viability of HemECs was significantly reduced by knock-down of LncRNA MCM3AP-AS1 (Figure 2A). We further analyzed whether LncRNA MCM3AP-AS1 induces apoptosis in HemECs. FACS analysis was employed to estimate the effect of silence of LncRNA MCM3AP-AS1 on the apoptosis in HemECs. The results demonstrated that the apoptotic ratio of HemECs was significantly increased after knock-down of LncRNA MCM3AP-AS1 (Figure 2B). To examine whether LncRNA MCM3AP-AS1 was linked to cell cycle arrest in HemECs, we analyzed the effects of knock-down of LncRNA MCM3AP-AS1 on cell cycle of HemECs, and found that the percentage of cells in the G1 phase was significantly increased (Figure 2C). The above results demonstrated that knockdown of LncRNA MCM3AP-AS1 inhibited proliferation, induced apoptosis of HemECs, and arrested hemangioma cells in the G1phase of cell cycle.

### LncRNA MCM3AP-AS1 Directly Targeted miR-138-5p

**FIGURE 3:**
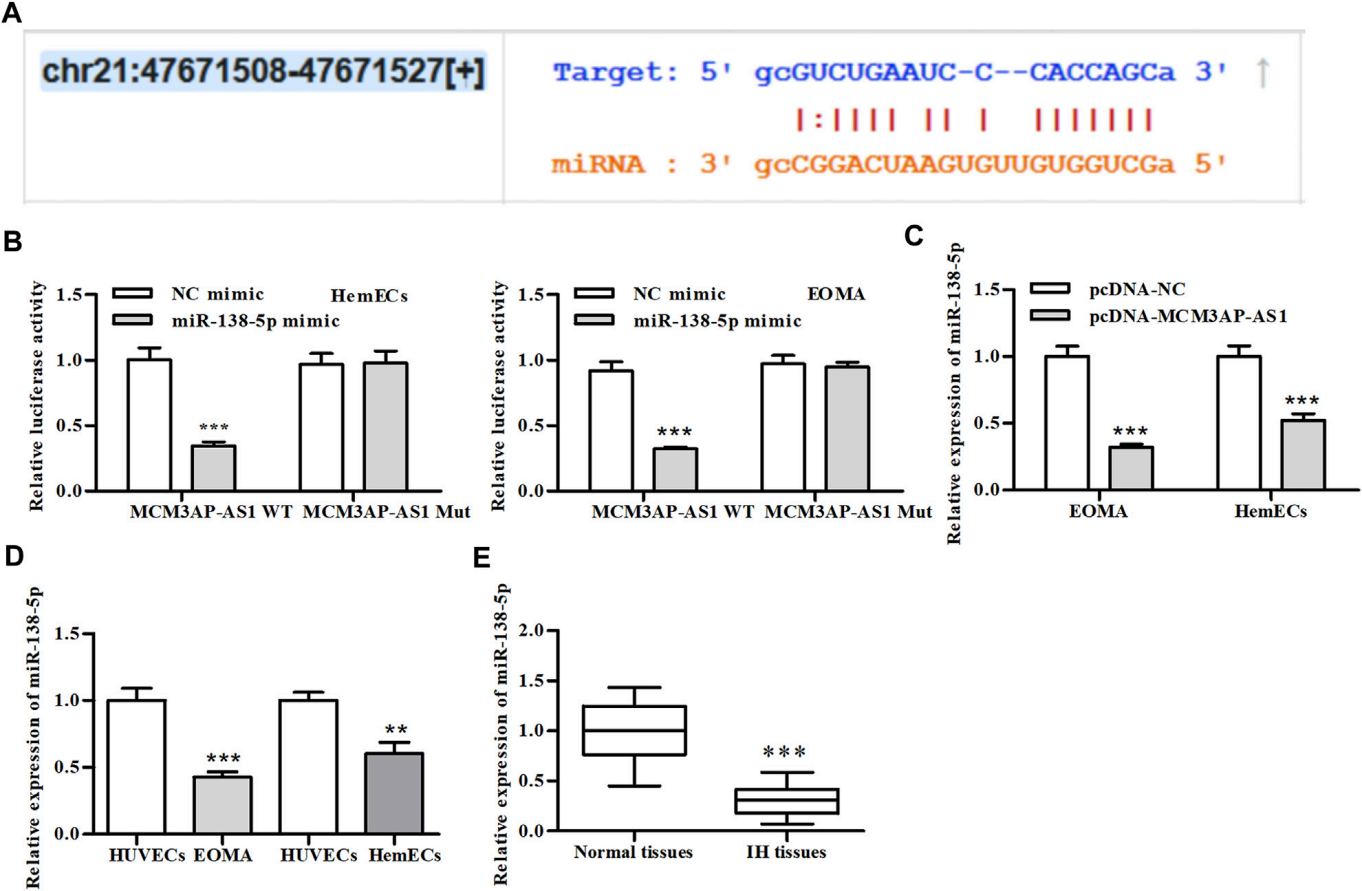
LncRNA-MCM3AP-AS1 directly binds to miR-138-5p. **(A)** The putative LncRNA-MCM3AP-AS1 binding sequence of miR-138-5p. **(B)** Luciferase activity of a luciferase reporter plasmid containing MCM3AP-AS1-WT or MUT co-transfected with miR-138-5p mimics or NC mimics by the dual luciferase reporter assay (***p < 0.001, compared with the NC mimic group). **(C)** The expression of miR-138-5p in MCM3AP-AS1-transfected HemECs cells (***p < 0.001, compared with the pcDNA-NC group, *n* = 3). **(D)** The expression of miR-138-5p compared between HemECs cell and HUVECs (**p < 0.01, ***p < 0.001, compared with the HUVECs group, *n* = 3). **(E)** The expression of miR-138-5p compared between IH patient tissue and normal skin tissue (***p < 0.001, compared with the Normal tissues group, *n* = 3).

To explore which miRNAs functioned as the sponge of LncRNA MCM3AP-AS1 to co-regulate the expression of downstream genes, we searched the starBase v3.0 (http://starbase.sysu.edu.cn/index.php) and found that LncRNA MCM3AP-AS1 harbored a putative binding site for miR-138-5p (Figure 3A). To further evaluate this conjecture, we construct the LncRNA MCM3AP-AS1-WT or LncRNA MCM3AP-AS1-Mut luciferase reporter and transfected these reporters into HemECs together with miR-138-5p mimic or its NC mimic. Dual-luciferase reporter assays showed that miR-138-5p mimic significantly reduced the luciferase activity of LncRNA MCM3AP-AS1-WT reporter, whereas exhibited modest effects on the activity of LncRNA MCM3AP-AS1-MUT reporter (Figure 3B). qPCR results showed that miR-138-5p mimics significantly reduced the expression of LncRNA MCM3AP-AS1 (Figure 3C). Then we detected the expression of miR-138-5p in HemECs and IH clinical tissues. As expected, the expression of miR-138-5p was significantly decreased in both IH cells and clinical samples compared with control groups (Figures 3D,E). These results demonstrated that LncRNA MCM3AP-AS1 directly targeted miR-138-5p*.*

### MiR-138-5p Targeted HIF-1α Directly

**FIGURE 4:**
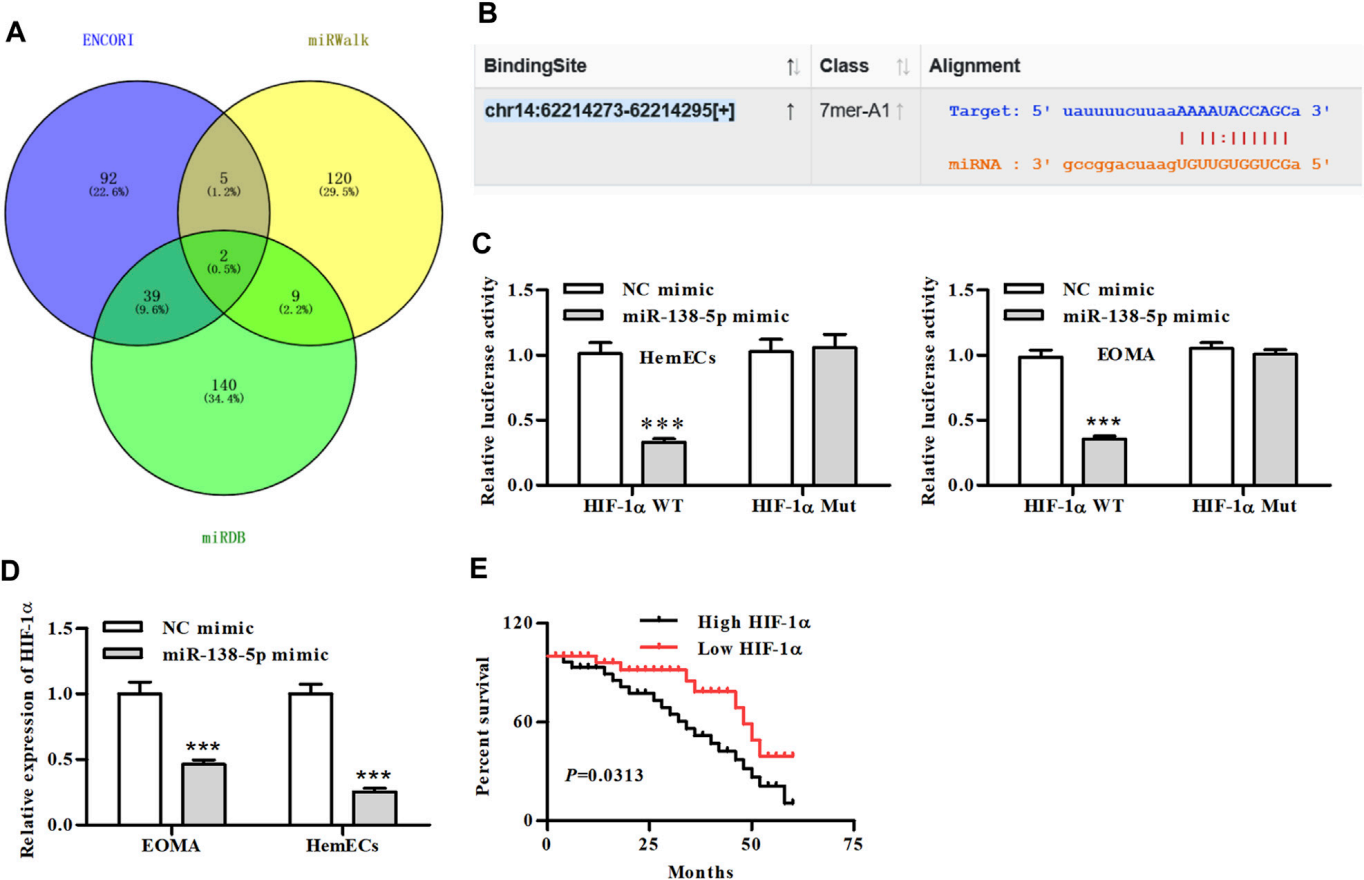
MiR-138-5p directly binds to HIF-1α. **(A)** Venn diagram of predicted genes screened from the three database **(B)** The putative miR-138-5p binding sequence of HIF-1α. **(C)** Luciferase activity of a luciferase reporter plasmid containing HIF-1α-WT or MUT co-transfected with miR-138-5p mimics or NC mimics by the dual luciferase reporter assay (***p < 0.001, compared with the NC mimic group). **(D)** The expression of HIF-1α in miR-138-5p-transfected HemECs cells (***p < 0.001, compared with the NC mimic group). **(E)** Kaplan-Meier analysis of OS compared high- and low-expression of HIF-1α in IH patients.

We then predicted the downstream target genes of miR-138-5p using the three databases and Venn analysis showed two overlapping genes (Figure 4A). Through further binding site analysis, we found that the downstream regulatory gene of miR-138-5p was the hypoxia-inducible factor-1alpha (HIF-1α) (Figure 4B). HIF-1α-WT and HIF-1α--Mut luciferase reporters were constructed and transfected into HemECs together with miR-138-5p mimic or its NC mimic. Dual-luciferase reporter assays showed that miR-138-5p mimic significantly reduced the luciferase activity of HIF-1α-WT reporter, whereas exhibited no significant effects on the activity of HIF-1α-MUT reporter (Figure 4C). qRT-PCR results showed that miR-138-5p mimics significantly reduced the expression of HIF-1α (Figure 4D). Further analysis of clinical patient information from the database demonstrated that patients with low expression of HIF-1α had significantly better overall survival (Figure 4E).

### The Process of Glycolysis in IH was Mediated by LncRNA MCM3AP-AS1 via Regulating miR-138-5p/HIF-1α Axis

**FIGURE 5:**
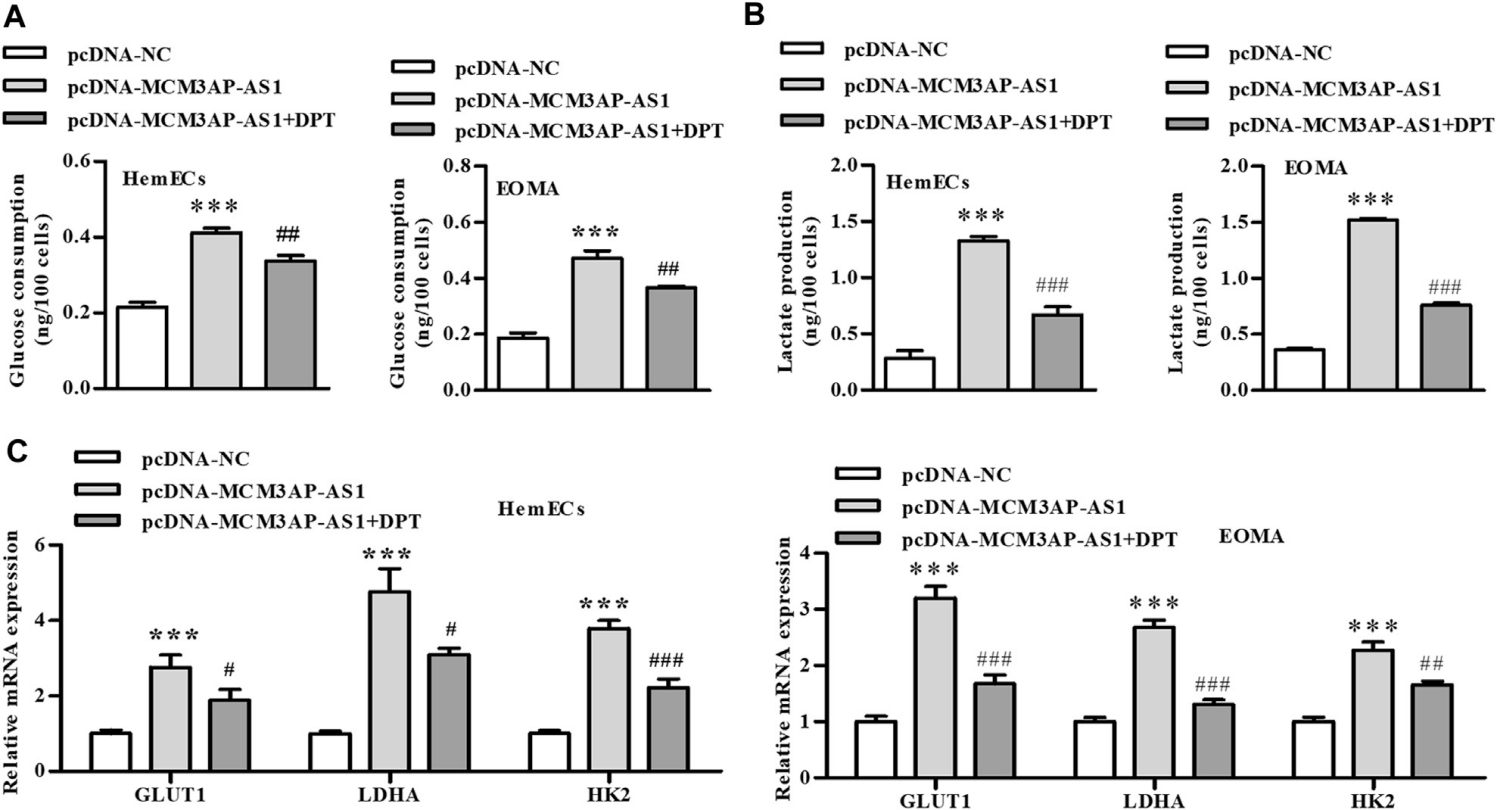
LncRNA-MCM3AP-AS1 mediates glycolysis in IH disease by regulating HIF-1α. **(A)** Glucose consumption test demonstrated that LncRNA MCM3AP-AS1 significantly increased glucose consumption of HemECs, and this elevated effect was partially reversed by adding HIF-1α inhibitor (DPT) (***p < 0.001 compared with the pcDNA-NC group; ^##^ *p* < 0.01 compared with the pcDNA-MCM3AP-AS1 group). **(B)** Lactate production test showed LncRNA MCM3AP-AS1 significantly increased lactate production of HemECs, and this elevated effect was partially reversed by adding HIF-1α inhibitor (DPT) (***p < 0.001 compared with the pcDNA-NC group; ^###^ *p* < 0.001 compared with the pcDNA-MCM3AP-AS1 group). **(C)** The expression of key genes involved in glycolysis. LncRNA MCM3AP-AS1 significantly increased the expression of GLUT1, LDHA and HK2, and this elevated effect was partially reversed by adding HIF-1α inhibitor (DPT) (***p < 0.001 compared with the pcDNA-NC group; ^#^ *p* < 0.05, ^##^ *p* < 0.01, ^###^ *p* < 0.001 compared with the pcDNA-MCM3AP-AS1 group).

Previous studies have shown that the abnormally elevated glycolysis level of tumor cells was reversed by inhibiting the expression of HIF-1α, which forced tumor cells to return to the metabolic mode of glucose oxidative phosphorylation, increased the energy consumption of tumor cells, and induced the death of tumor cells. Therefore, we examined the effect of HIF-1α on glycolysis in IH. Our results showed that LncRNA MCM3AP-AS1 significantly increased glucose consumption of HemECs, but this effect was partially reversed by adding HIF-1α inhibitor (DPT) (Figure 5A). Since the abnormally increased glycolysis of tumor cells was mainly manifested as increased pyruvate production and lactic acid production, we also tested the effect of HIF-1α on lactic acid production in IH. Results revealed that LncRNA MCM3AP-AS1 significantly increased lactic acid production of HemECs, but this effect was partially reversed by adding HIF-1α inhibitor (DPT) (Figure 5B). We also examined the expression of several key genes involved in glycolysis. The qPCR results showed that LncRNA MCM3AP-AS1 largely increased the expression of GLUT1, LDH and HK2, while HIF-1α inhibitor (DPT) partially reverse the enhancement of these genes expression induced by LncRNA MCM3AP-AS1 on IH (Figure 5C).

**FIGURE 6:**
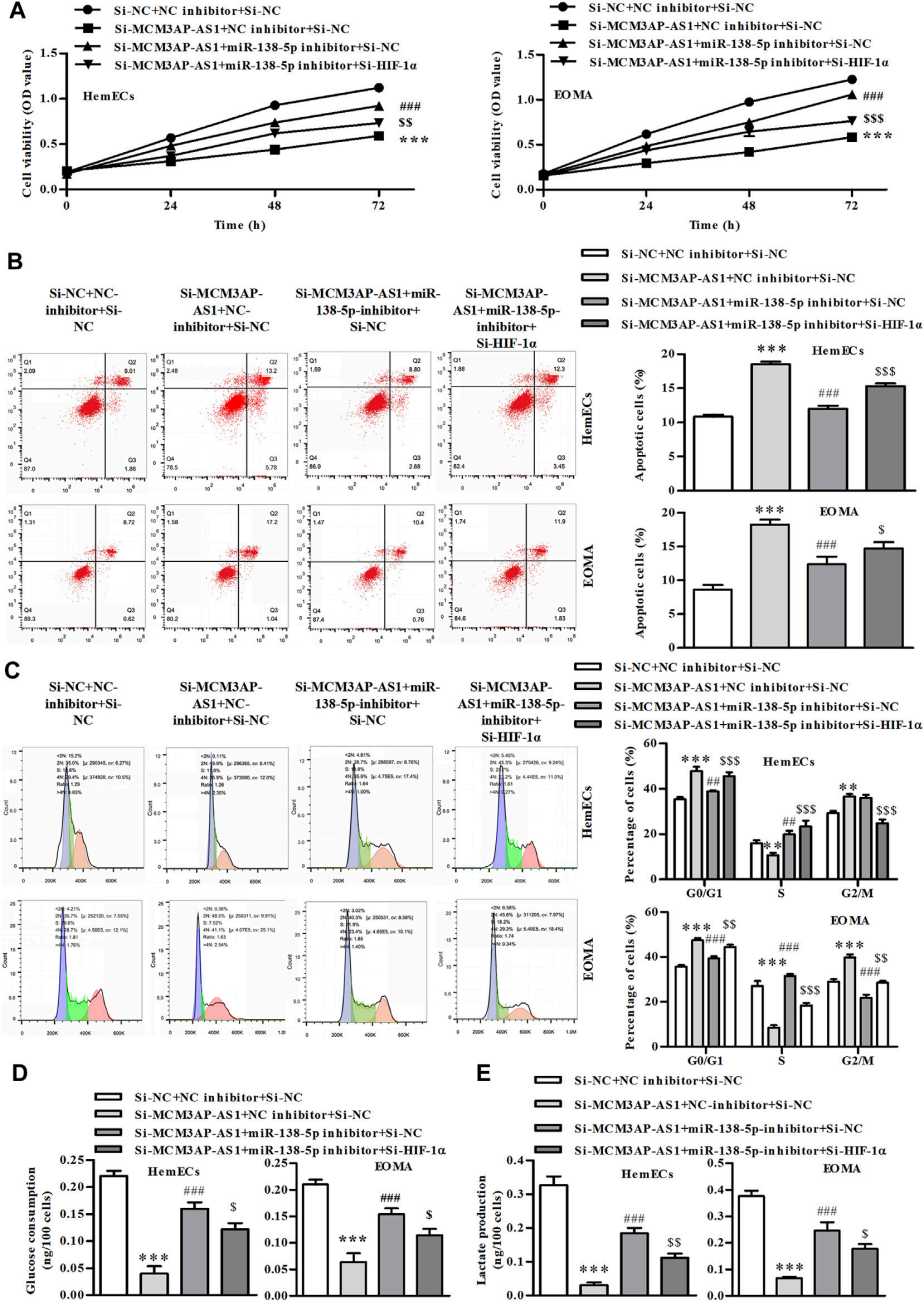
The process of glycolysis in IH is mediated by LncRNA MCM3AP-AS1 via regulating miR-138-5p/HIF-1α axis. **(A)** In CCK-8 assay on HemECs treated with NC mimics, si-MCM3AP-AS1, miR-138-5p, or si-HIF-1α 24, 48 and 72 h. **(B)** FACS analysis showing the apoptosis induced by NCmimics, siMCM3AP-AS1, miR-138-5p or siHIF-1α 48 h in HemECs. **(C)** Cell cycle analysis indicating cell cycle arrested by NCmimics, siMCM3AP-AS1, miR-138-5p or siHIF-1α in HemECs. **(D)** Glucose consumption test demonstrated that the effects of NCmimics, siMCM3AP-AS1, miR-138-5p or siHIF-1α on the HemECs. **(E)** Lactate production test demonstrated that the effects of NCmimics, siMCM3AP-AS1, miR-138-5p or siHIF-1α on the HemECs (**p < 0.01, ***p < 0.001 compared with the si-NC + NC inhibitor + si-NC group; ^##^ *p* < 0.01, ^###^ *p* < 0.001 compared with the si-MCM3AP-AS1 + NC inhibitor + si-NC group; ^$^ *p* < 0.05, ^$$^ *p* < 0.01, ^$$$^ *p* < 0.001 compared with the si-MCM3AP-AS1 + miR-138-5p inhibitor + si-NC group).

We further evaluated whether LncRNA MCM3AP-AS1 exerted regulatory roles via miR-138-5p/HIF-1α pathway in HemECs. CCK-8 analysis demonstrated that LncRNA MCM3AP-AS1 knockdown alone decreased the number of HemECs, while miR‐138-5p inhibitor alone or siHIF-1α significantly attenuated the proliferation-inhibiting effects of siLncRNA MCM3AP-AS1 on HemECs (Figure 6A). Similarly, the FACS analysis results demonstrated that the apoptotic ratio of HemECs was significantly increased after knock-down of LncRNA MCM3AP-AS1. However, miR‐138-5p inhibitor alone or siHIF-1α significantly reversed the apoptosis-inducing effects of siLncRNA MCM3AP-AS1 on HemECs (Figure 6B). The cell cycle analysis revealed that most HemECs were arrested at G0/G1phase of the cell cycle in cells with LncRNA MCM3AP-AS1 knock-down. Interestingly, miR‐138-5p inhibitor alone or siHIF-1α partially reversed the effect induced by siLncRNA MCM3AP-AS1 on HemECs (Figure 6C). Further analysis showed that knock-down of LncRNA MCM3AP-AS1 significantly decreased the glucose consumption and lactate production while miR‐138-5p inhibitor alone or siHIF-1α significantly reversed these effects of siLncRNA MCM3AP-AS1 on HemECs (Figures 6D,E).

### LncRNA MCM3AP-AS1, miR‐138-5p and HIF-1α Expressed in IH Tissues

**FIGURE 7:**
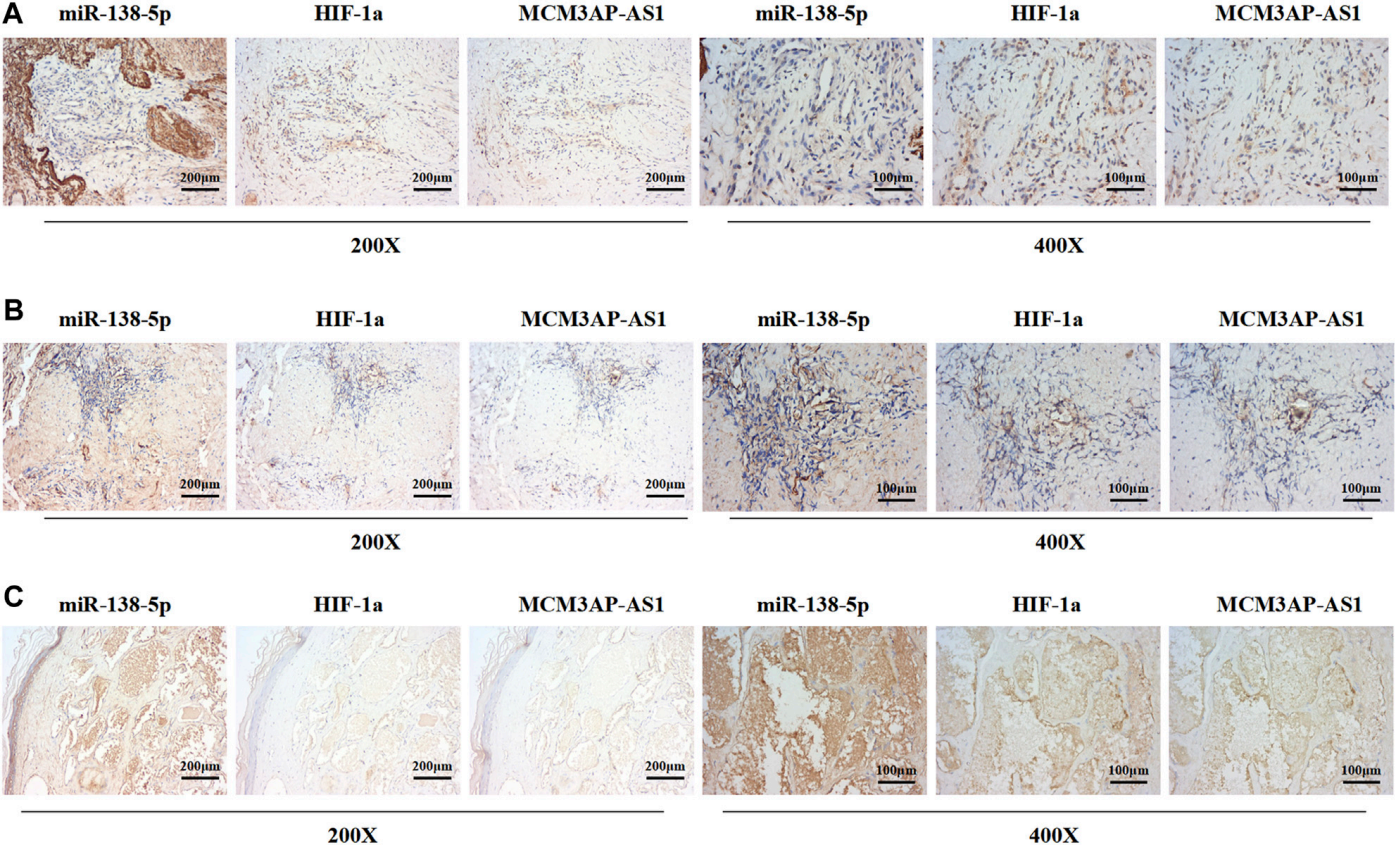
The expression of LncRNA MCM3AP-AS1 is negatively correlated with miR‐138-5p, and miR‐138-5p negatively correlated with HIF-1α in IH tissues. **(A–C)** ISH and IHC result from three IH clinical tissues. The ISH score of miR-1306-3p is negatively correlated with LncRNA MCM3AP-AS1 in the IH tissue. The ISH score of miR-1306-3p is negatively correlated with the IHC scores of HIF-1α in the IH tissues.

Previous results have demonstrated that LncRNA MCM3AP-AS1 and miR-138-5p played an opposite role in the progression of IH disease, and miR-138-5p negatively regulates HIF-1α expression. Therefore, we examined the expression of these three genes in clinical tissues. IHC was employed to evaluate the expression of LncRNA MCM3AP-AS1 on the same ISH which was used for the evaluation of miR-138-5p expression. We found that both of LncRNA MCM3AP-AS1 and miR-138-5p were expressed in three clinical tissues. The same analysis strategy was used to examine the expression of miR-138-5p and HIF-1α (Figures 7A-C). The above analysis demonstrated that the expression of LncRNA MCM3AP-AS1 is negatively correlated with miR‐138-5p, and miR‐138-5p negatively correlated with HIF-1α in IH tissues.

## Discussion

Infantile hemangioma is the most common childhood tumor, generally affecting the head and neck region. The tumor may grow rapidly and cause obstruction in normal anatomical structures, leading to severe damage. As a class of non-coding RNAs that has attracted a lot of attention in recent years, LncRNA plays important roles in the process of tumor development, proliferation and metastasis (Gu et al., 2018). MCM3AP-AS1 is the antisense LncRNA of MCM3AP, which is associated with the malignant progression of various tumors such as thyroid cancer and liver cancer (Liang et al., 2019; Zhang et al., 2019). Wang et al. (2019c) found that high expression of MCM3AP AS1 was positively correlated with large tumor volume, high tumor grade, advanced tumor stage and poor prognosis of HCC patients. Yang et al. (2019) indicated that silencing of MCM3AP-AS1 inhibited the proliferation of pancreatic cancer cells. In addition, previous studies have reported that LncRNAs displayed crucial roles during IH progression. Liu et al. (2019a) demonstrated that knock down of LncRNA LINC00342 inhibited the proliferation of infantile hemangioma by sponging miR-3619-5p. Zhang and Zhang (2019) and his colleagues found that LncRNA UCA1 was highly expressed in IH, and LncRNA UCA1 promoted proliferation, migration and invasion via regulating miR-200c in hemangioma cells. In this study, we found that MCM3AP-AS1 was highly expressed in IH cell lines and clinical specimens from IH patients. Moreover, IH patients with highly expressed MCM3AP-AS1 had a worse survival rate.

In this study, we confirmed that LncRNA MCM3AP-AS1 was highly expressed in IH clinical tissue samples and IH cells, and patients with high expression of LncRNA MCM3AP-AS1 had a worse survival rate. Importantly, knock down of LncRNA MCM3AP-AS1 significantly inhibited the proliferation of IH cells, and arrested the cell cycle at G1 phase. Furthermore, by bioinformatic and experimental analysis, we found that LncRNA MCM3AP-AS1 bound to miR-138-5p, and the downstream target gene of miR-138-5p was HIF-1α. In addition, we observed that the process of glycolysis in IH was mediated by LncRNA MCM3AP-AS1 via regulating miR-138-5p/HIF-1α axis, and confirmed this phenomenon through the rescue experiment. At last, we found that LncRNA MCM3AP-AS1 negatively correlated with miR‐138-5p, and miR‐138-5p negatively correlated with HIF-1α in IH patients. Based on the above results, we demonstrated that knock down of LncRNA-MCM3AP-AS1 inhibits the progression of hemangioma by decreasing glycolysis via regulating miR-138-5p/HIF-1α axis, which laid a foundation for exploring the molecular mechanism of IH treatment.

MicroRNA-138-5p is one of the important tumor suppressor regulators, and has been found in breast cancer (Xun et al., 2021), gastric cancer (Fan et al., 2021), renal cell carcinoma (Xue et al., 2021), pancreatic cancer (Han et al., 2021), and other tumors. In recent studies on breast cancer (Xun et al., 2021), it has been found that miR-138-5p mimics can be transfected to up-regulate miR-138-5p which reduced the expression of KDM6B and thus inhibited M1 polarization and promoted M2 polarization of macrophages in breast cancer. Fan et al. found that miRNA was expressed at low level in patients with gastric cancer (Fan et al., 2021), and knock down of Circ-CORO1C inhibited the proliferation of gastric cancer by sponging miR-138-5p. More importantly, the down-regulation of miR-138-5p was of special significance for lymph node metastasis and local infiltration of gastric cancer. In the study of pancreatic cancer (Han et al., 2021), it was found that miR-138-5p was down-regulated in PANC-1 cells, and silencing of miR-138-5p induced the increase of CCAT1 and HMGA1 expression. CCAT1 competitively bound to miR-138-5p to regulate downstream HMGA1 expression, and ultimately affected the progression of pancreatic cancer. However, to the best of our knowledge, there is no existing research on how miR-138-5p affected the proliferation, apoptosis of IH cells and survival rates of IH patients. In this study, by bioinformatics analysis, we found that LncRNAs MCM3AP AS1 bound to miR-138-5p, and the binding was verified by dual luciferase reporter assay and qPCR. More importantly, our results showed that miR-138-5p inhibited the proliferation of IH cells by targeting HIF-1α.

Hypoxia is one of the characteristics of most tumors. Hypoxic microenvironment exists in the vast majority of tumors, including liver cancer (Ma et al., 2021a) and IH (Wu et al., 2021). The hypoxic microenvironment promotes the metastasis of tumor cells (Yu et al., 2021), enhances the tolerance of tumor cells to radiotherapy and chemotherapy (Sun et al., 2021), and also changes the gene expression of tumor cells. HIF-1α is an important transcription factor in hypoxic microenvironment. It is strictly regulated by oxygen concentration and is abnormally overexpressed in hypoxic tissues (Chappell et al., 2019). HIF-1α plays a regulatory role of hypoxia on tumor cell genes and promotes the survival of tumor cells in hypoxic microenvironment (Gonzalez et al., 2018). In the present study, we not only found that miR-138-5p mimics reduced the expression of HIF-1α in IH disease, but also that IH patients with low expression of HIF-1α had a better survival rate, suggesting that HIF-1α promotes the progression of IH, and these findings are consistent with the results of previous studies.

Previous studies have shown that one common phenomena in the development of tumors is hypoxia (Li et al., 2021; Liu et al., 2021; Zhu et al., 2021). In the process of tumor growth, due to the active growth of tumor cells, the degree of proliferation exceeds the speed of angiogenesis, resulting in local tissue hypoxia (Liu et al., 2021). Therefore, the adaptation of cells to hypoxia is a key step in tumor pathogenesis, and its main mechanisms are glucose transportation, glycolysis, and tumor angiogenesis (Ma et al., 2021b). Under hypoxic condition, the expression of HIF-1α increase exponentially (Peng et al., 2014). HIF-1α is up-regulated in almost all types of tumors and is involved in initiating transcription of several genes involved in tumor growth adaptation to anoxic environment. There are more than 60 target genes of HIF-1α (Hepp et al., 2021; Quiroga et al., 2021), such as erythropoietin (EPO), glucose transport protein-1 (Glut-1), vascular endothelial growth factor (VEGF), tyrosine hydroxylase, glycolysis related enzymes and more. HIF-1α induces the expression of these factors and enzymes and induces a series of hypoxia adaptation responses in the body. We confirmed that upregulation of LncRNA MCM3AP AS1 leads to increased glucose consumption and lactic acid production in IH patients, which is manifested by increased glycolytic levels in IH patients. We also demonstrated that inhibition of HIF-1α in IH patients can partially reverse this elevated effect caused by upregulated LncRNAs MCM3AP AS1.

It was known that LncRNAs play important roles in tumors by targeting and regulating mRNA through competitive binding to miRNAs (Chou et al., 2016). For example, MCM3AP-AS1 promotes the progression of papillary thyroid carcinoma by regulating the miR-211-5p/SPARC axis (Liang et al., 2019). In liver cancer, LINC-RORs up-regulate the expression of FOXMI by adsorbing miR-876-5p, thereby promoting the proliferation and metastasis of tumor cells (Zhi et al., 2019). In CRC, LncRNA GAS5 inhibits the proliferation of cancer cells and promotes apoptosis by targeting miR-222-3p (Liu et al., 2019b). In this study, we proved that the process of glycolysis in IH was mediated by LncRNA MCM3AP-AS1 via regulating miR-138-5p/HIF-1α axis. Further analysis on clinical specimens demonstrated that the expression of LncRNA MCM3AP-AS1 was negatively correlated with miR‐138-5p, and miR‐138-5p negatively correlated with HIF-1α in IH patients.

In summary, this study demonstrated that LncRNA-MCM3AP-AS1 promotes the progression of hemangioma by regulating miR-138-5p/HIF-1α axis and glycolysis. This study suggested the potential value of LncRNA-MCM3AP-AS1/miR-138-5p as a treatment strategy for hemangioma.

## Materials and Methods

### Clinical Samples

Twenty-two infantile hemangioma specimens including normal subcutaneous tissues and infantile hemangioma tissues in the involuting stage (four males and eight females; median age, 7 months) and proliferating stage (two males and eight females; median age, 6 months), were collected in the plastic surgery department of our hospital from October 2019 to October 2020. All the samples were immediately frozen at −80°C for further analysis. The informed consent was obtained from the parents of patients. The experiments were approved by the Ethics Committee for Experiment of Nantong University.

HemECs were isolated from IH tissues in the proliferating phase as previously described (Khan et al., 2006). HemECs were cultured in human endothelial-serum free medium (Gibco; Thermo Fisher Scientific, Inc.) containing 10% FBS (Gibco; Thermo Fisher Scientific, Inc.) with 5% CO_2_ at 37°C.

### RNA Extraction and Quantitative Real-Time PCR

All the specimens were detached quickly and immersed in lysis solution (4305895, Thermo Scientific) at the ratio of 1:6, then the samples were homogenized using an ultrasound homogenizer. Total RNA extraction was performed as the supplier’s protocol (AM 1912, Thermo Scientific). NanoDrop ND-1000 spectrophotometer (Thermo Scientific) was used to evaluate the quantity and purity of RNA. Complementary DNAs were obtained by the reverse transcription reaction with a reverse transcription kit (RR037A, Takara) and applied as templates to determine the expression of target genes by PCR. All the primers were designed by Primer Premier 5.0 software, and synthesized by Sangon Biotech (Shanghai, China). The primers were designed and synthesized as follows: hsa-miR-138-5p, 5′-gtc​gta​tcc​agt​gca​ggg​tcc​gag​gta​ttc​gca​ctg​gat​acg​acc​ggc​ct-3′; LncRNA-MCM3AP-AS1, forward: 5′-GCT​GCT​AAT​GGC​AAC​ACT​GA-3′ and reverse: 5′-AGGTGCTGTCTG GTGGAGAT-3′. GAPDH, forward: 5′-CAG GAG​GCA​TTG​CTG​ATG​AT-3′ and reverse: 5′-GAA​GGC​TGG​GGC​TCA​TTT-3′. LncRNA H19 forward: 5′-ACC​ACT​GCA​CTA​CCT​GAC​TC-3′, and reverse: 5′-CCG​CAG​GGG​GTG​GCC​ATG​AA-3′; LncRNA AC092718.4 forward: 5′-GCA​ACT​CCT​AGA​TTC​GAT​AC-3′, and reverse: 5′-CAC​GCG​TAT​GGT​AAC​ATG​CT-3′; LncRNA NEAT1 forward: 5′-TTCCGTGCT TCCTCTTCTGT-3′ and reverse: 5′-CAG​GGT​GTC​CTC​CAC​CTT​TA-3′; LncRNA PARD6G-AS1, forward: 5′-ATG​CTG​CAA​CTT​GTA​ACT-3′ and reverse: 5′-ACT​TGC​GAC​TTG​ACA​CTT​AGA​TT-3′;

### Cell Counting Kit-8 Assay

CCK-8 assay was used to assess the cell viability of HemECs and EoMA cells. Briefly, HemECs and EoMA cells (about 10,000 per well) were seeded into 96-well plates. After 24, 48, and 72 h, CCK-8 solution (10 μL) was added into each well and incubated with HemECs and EoMA cells at 37 °C for 3 h. Then the light absorbance was measured at the wavelength of 450 nm to assess the cell viability of HemECs and EoMA cells.

### Flow Cytometric Analysis of Cell Cycle Distribution and Apoptosis

The cells were seeded into 6-well plates. For cell cycle analysis, the cells were transfected with the different plasmids for 48 h. Then, the cells were washed by PBS (4°C), and collected with cold 70% ethanol (4°C) followed by washing with PBS. After the above operations, the cells were incubated with 1 ml of 20 mg/ml propidium iodide (PI) which contained RNase (1 mg/ml) in PBS for 30 min followed by fluorescence-activated cell sorting (FACS) assay. For the apoptosis assay, the cells were collected after treatment with the different plasmids for 48 h. The cells were washed with cold (4°C) PBS, followed by incubation with PI and Annexin V-EGFP according to the procedures specified in the kit (KeyGen Biotech Co. Ltd., Nanjing, China). Then, the processed cells were inspected using FACS assay.

### Dual‐Luciferase Reporter Assay

For the luciferase reporter assay, a dual luciferase reporter assay system (Promega corporation) was used to detect the binding between miR-138-5p and LncRNA MCM3AP-AS1 or 3′-UTR of HIF-1α according to the manufacturer’s protocols. HemECs were seeded onto a 6-well plate and cultured for 24 h, cells were co-transfected with pGL3-MCM3AP-AS1 WT, or pGL3-MCM3AP-AS1 Mut (pGL3-HIF-1α 3′UTR WT or pGL3-HIF-1α 3′UTR Mut) and miR-138-5p mimic/NC mimic. After 48 h, luciferase activity was detected using the Dual-Luciferase Reporter Assay System (Promega, Madison, WI, United States).

### *In situ* Hybridization

The paraffin-embedded tissues were cut into 4 μm thick sections and sections heated at 56°C for 5 min. After that, xylene and ethanol were used for deparaffinization. The sections were sealed with 3% H_2_O_2_ for 10 min, hybridized with 20 μl pre hybridizing solution in 42°C incubators for 3.5 h, and hybridized with 20 μl probe (The corresponding probe for detecting genes) solution (synthesized by Sangon Biotech Co., Ltd., Shanghai, China) in 40°C incubators for 20 h. The hybridization was coated with 3% BSA for 2 h in 37°C, washed and sealed. Biotinylated secondary anti-digoxin, streptavidin-biotin peroxidase complex ABC and DAB were added in turn according to the instructions of the ISH kit. Finally, the samples were observed under a light microscope.

### Immunohistochemical Analysis

As for immunohistochemical analysis, paraffin-embedded tissue sections were deparaffinized by xylene and hydrated with gradient ethanol. Then, the sections were treated with citrate buffer (pH = 6) for 20 min and immersed in 3% H_2_O_2_ for 10 min in a humidified chamber. After washing with PBS 5 min 3 times and blocked for 30 min with 10% goat serum, sections were incubated with rabbit HIF-1α antibody (1:400 Abcam, Cambridge, MA, United States) in a humidified chamber at 4°C overnight with a two-step protocol. Five randomly selected fields were captured under high-power magnification (×200) using a bright-field microscope (Olympus, Tokyo, Japan) and analyzed using Image-Pro Plus v6.2 software (Media Cybernetics, Silver Spring, MD).

### Binding Sites Prediction

The ENCORI database (http://starbase.sysu.edu.cn/), miRWalk database (http://mirwalk.umm.uni-heidelberg.de/) and miRDB database (http://www.mirdb.org) are powerful databases to study non-coding RNAs, such as LncRNAs, miRNAs and circRNAs. These databases were used to predict the binding sites between LncRNA MCM3AP-AS1 and miR-138-5p or miR-138-5p and the 3′-UTR of HIF-1α.

### Statistical Analysis

All the experiments were repeated at least three times independently. Data were analyzed using SPSS (SPSS 12.0, SPSS (IBM) Inc., Illinois, United States). The categorical variants were assigned a numerical value. Data were presented as mean ± standard deviation. Unpaired Student’s t tests or one-way ANOVA was used for data comparison (SPSS 12.0, SPSS (IBM) Inc., Illinois, United States). Statistical significance was determined at defined *p* < 0.05.

## Data Availability

The original contributions presented in the study are included in the article/Supplementary Materials, further inquiries can be directed to the corresponding author.
